# Quinine Intoxication

**Published:** 1890-08

**Authors:** 


### QUININE INTOXICATION.

Dr. Lewis A. Sayre, of New York, says that there are many cases on record where the use of quinine has caused a disarrangement of the mental powers, and to such an extent that the sufferer did not know what he or she was about. Instances are not few where patients who were given large doses of the drug became delirious. These symptoms, however, passed away when the use of quinine was discontinued. It is possible while under its influence for one to act as irresponsibly as when in liquor. That quinine affects the brain is evident from the fact that an overdose will cause severe buzzing in the ears, and often temporary deafness.

Physicians cannot be too careful in prescribing quinine, for what is one man's meat is another man's poison. I have known one grain to have more effect on some patients than fifteen grains on others. The same can be said of morphine. Two grains of this drug will cause many intense itching sensations, with parched tongue and throat. On the other hand, I have known patients, even those used to morphine, to take much larger doses without showing any evil effects. There is little doubt, but there are quinine habitues as well as slaves to chloral, morphine and other narcotics and drugs, yet its use as a stimulant has not become general.

